# Leveraging Computational Intelligence Techniques for Diagnosing Degenerative Nerve Diseases: A Comprehensive Review, Open Challenges, and Future Research Directions

**DOI:** 10.3390/diagnostics13020288

**Published:** 2023-01-12

**Authors:** Saransh Bhachawat, Eashwar Shriram, Kathiravan Srinivasan, Yuh-Chung Hu

**Affiliations:** 1 School of Computer Science and Engineering, Vellore Institute of Technology, Vellore 632014, India; 2 School of Information Technology and Engineering, Vellore Institute of Technology, Vellore 632014, India; 3 Department of Mechanical and Electromechanical Engineering, National Ilan University, Yilan 26047, Taiwan

**Keywords:** degenerative nerve diseases, neurodegenerative disorder, machine learning, deep learning, progressive brain diseases, diagnosis

## Abstract

Degenerative nerve diseases such as Alzheimer’s and Parkinson’s diseases have always been a global issue of concern. Approximately 1/6th of the world’s population suffers from these disorders, yet there are no definitive solutions to cure these diseases after the symptoms set in. The best way to treat these disorders is to detect them at an earlier stage. Many of these diseases are genetic; this enables machine learning algorithms to give inferences based on the patient’s medical records and history. Machine learning algorithms such as deep neural networks are also critical for the early identification of degenerative nerve diseases. The significant applications of machine learning and deep learning in early diagnosis and establishing potential therapies for degenerative nerve diseases have motivated us to work on this review paper. Through this review, we covered various machine learning and deep learning algorithms and their application in the diagnosis of degenerative nerve diseases, such as Alzheimer’s disease and Parkinson’s disease. Furthermore, we also included the recent advancements in each of these models, which improved their capabilities for classifying degenerative nerve diseases. The limitations of each of these methods are also discussed. In the conclusion, we mention open research challenges and various alternative technologies, such as virtual reality and Big data analytics, which can be useful for the diagnosis of degenerative nerve diseases.

## 1. Introduction

Degenerative nerve diseases, or neurodegenerative disorders, are diseases caused due to the gradual degeneration of neurons so that the connection between the cells and the nervous system weakens. These disorders are considered incurable and are usually observed in the elderly, i.e., at ages above 60. With the increase in population, the number of patients suffering from degenerative nerve diseases is increasing exponentially. The World Health Organization reported that, in 2022, more than 55 million people were diagnosed with dementia and over 60% of these patients live in low- or middle-income countries [1]. This means that a vast majority of these patients might be unaware of the early symptoms and are unable to obtain early treatment. The number of patients with dementia is increasing at a rate of 10 million per year. This rapid rate in cases of dementia as well as other neurodegenerative disorders makes them a major concern for society. Therefore, there has also been a significant increase in research aimed at diagnosing patients at an early stage of the disease so that its progression can be slowed down [2,3,4]. Some of the most common degenerative nerve diseases include Alzheimer’s disease, Parkinson’s disease, dementia, Huntington’s disease, and amyotrophic lateral sclerosis, with Alzheimer’s being the most common. Their usual effects include loss of memory, irregular behavior, and damage to the functioning of motor neurons.

Machine learning (ML) and deep learning (DL) show a lot of potential for the diagnosis of degenerative nerve diseases [5,6]. In the last decade, new technologies such as ultrasonography and magnetic resonance imaging (MRI) were created, enabling large datasets that can be studied by ML and DL algorithms [7,8]. Recently, datasets were enriched with data obtained from advanced technologies such as electroencephalography (EEG) and single-photon emission computerized tomography (SPECT), which have made model predictions even more accurate [9]. This boosted the number of researchers studying and creating new frameworks for the early diagnosis of degenerative nerve diseases. These models have shown better scalability and accuracy than their predecessors. One such model, VEPAD, uses a random forest classifier to identify the different harmful variants of Alzheimer’s disease at a very high accuracy [10]. Another novel model, which uses a convolutional recurrent neural network and cross dataset learning with an extreme learning machine to classify patients of Parkinson’s disease using intrinsic emotions, has shown great performance, giving better results than even state-of-the-art studies [11]. Thus, surveying these recent developments in ML and DL can give great insights into the future potential of using these models for the early diagnosis of degenerative nerve diseases. Table A1 in Appendix A contains a list of glossary/nomenclature/abbreviations used in this review, as well as their definitions.

### 1.1. Contributions of This Review

Our survey contributions can be summarized as follows:

- Through this paper, we present an extensive study of machine learning and deep learning models used for diagnosing degenerative nerve diseases and show that ML and DL have a high potential for facilitating the diagnosis of degenerative nerve diseases.
- We give a detailed account of the recent developments made to improve the accuracy, scalability, and sensitivity of various ML and DL algorithms mentioned in this paper.
- Key highlights and limitations of these newly developed models are also discussed in detail.
- Along with this, alternate research directions for the diagnosis of degenerative nerve diseases disorders using the fields of IoT (Internet of Things), quantum computing, Big data analytics, etc., are also touched upon.

### 1.2. Shortcomings of the Existing Reviews

There are numerous reviews on the diagnosis of degenerative nerve diseases using artificial intelligence. They cover the various ML and DL models and a variety of diseases, but a major shortcoming in many such reviews is the lack of an exhaustive study of both ML and DL models. A large number of reviews have also not explored other fields that have the potential to contribute to the early diagnosis of degenerative nerve diseases. Our article is a comprehensive review of the various new ML and DL models being developed, how they have been implemented, what their limitations are, and what the future scope of improvement is and other technology fields such as Internet of Things and digital twin, which can be used to further aid in the diagnosis of degenerative nerve diseases.

## 2. Survey Methodology

**Figure 1:**
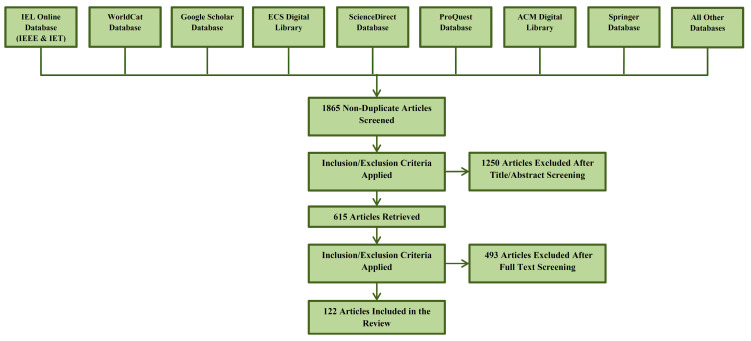
PRISMA flow diagram for the selection process of research articles used in this review: computational intelligence techniques for diagnosing degenerative nerve diseases.

**Figure 2:**
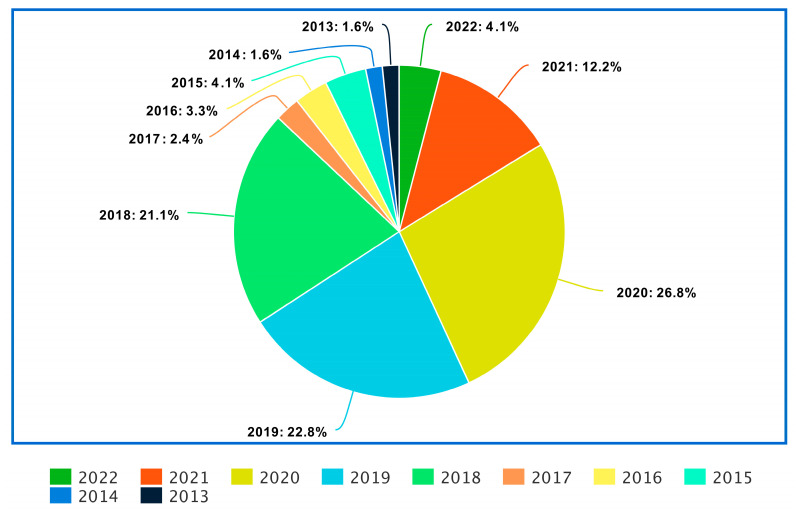
The distribution of the reviewed papers by year.

**Figure 3:**
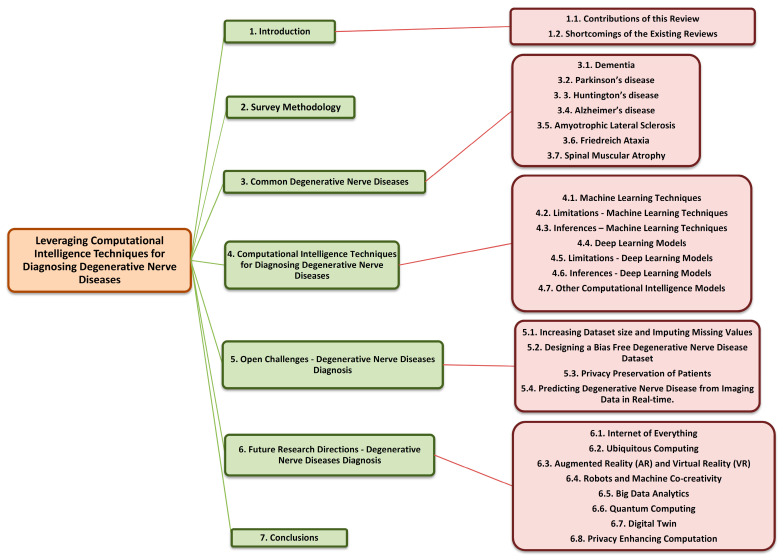
General structure of this review article.

For this survey, we utilized the Preferred Reporting Items for Systematic Reviews and Meta-Analyses (PRISMA) procedure to identify and choose which articles were relevant to this survey and filter those articles from the others. We sourced the articles from Google Scholar, ScienceDirect, IEEE Xplore, Elsevier, Springer, and other databases for this review. The referred articles include those written and published in English between January 2013 and February 2022 on various machine learning and deep learning algorithms and their applications for diagnosing degenerative nerve diseases. Articles that were published in languages other than English between January 2013 and February 2022, including case reports, opinions, commentaries, and dissertations, were excluded from this survey. After shortlisting the 1865 papers, we applied the exclusion and inclusion criteria and shortlisted 122 papers. Figure 1 portrays the PRISMA flow diagram for the selection process of the research articles used in this review. Figure 2 shows the distribution of the reviewed papers by year. Figure 3 shows the general structure of this review article.

## 3. Common Degenerative Nerve Diseases

Degenerative nerve diseases are neurological diseases that, with time, deteriorate the nervous functioning in our body, which causes cognitive impairments. Most of these diseases do not have a definitive cure, but detecting them at an early stage can help slow down the disease progression at a significant rate.

### 3.1. Dementia

Dementia is a degenerative nerve disease generally observed in the elderly, in which one person loses two or more cognitive abilities [12]. This is mainly due to some injury to the brain or some brain-related damage. The symptoms of the disease are cognitive impairment, hallucinations, both auditory and visual, and depression [13]. It is generally observed in people above the age of 65. With early diagnosis of this disease, we can slow down the process of this disease in our body to maintain the patient’s mental consciousness. Dementia contributes to about 10% of the total number of diseases observed in patients suffering from degenerative nerve diseases [14].

### 3.2. Parkinson’s Disease

Parkinson’s disease is the second most common degenerative nerve disease in the world. It also affects the elderly, and it is most common in the age group above 60. Medical help can help the patient a lot only if detected early in the patients [15]. Some of the common symptoms of Parkinson’s disease are a change in posture and change in gait, along with bradykinesia [16]. Until now, changes observed in the genes of *SNCA*, *LRRK2*, *VPS35*, *PRKN*, *PINK1*, *DJ-1*, and *GBA* have been linked with the typical PD in humans [17].

### 3.3. Huntington’s Disease

Huntington’s disease is related to a DNA strand called the CAG (cytosine, adenine, guanine) trinucleotide repeat. It is caused when this is dominantly inherited by the hunting gene on chromosome 4 of the human body [18]. This disease is characterized by behavioral symptoms, cognitive decline, and movement disorder [19]. Recent studies have shown that the mutated huntingtin protein in humans is not only found in the central nervous system but also found in different organs and tissues of the body [20].

### 3.4. Alzheimer’s Disease

Alzheimer’s disease is the most common degenerative nerve disease in humans. Currently, researchers are working on symptomatic therapy as a treatment for this disease, but medical help can significantly improve the quality of the person’s life only if detected in the early stages [21]. The symptoms are difficulties with problem-solving, language, and the memory of a person [22]. Right now, there are two classes of approved drugs to treat Alzheimer’s disease, which include inhibitors to the cholinesterase enzyme and antagonists to N-methyl D-aspartate, but these cannot cure or prevent the disease in humans [23].

### 3.5. Amyotrophic Lateral Sclerosis

Known also as ALS, this is a degenerative nerve disease that also affects the motor system of a human being. The person experiences weakness most commonly in the limb and distal muscles [24]. Generally, it proves fatal within 2–5 years of its onset in an individual. Its symptoms are upper and lower motor neuron degeneration [25]. A variety of cellular-level processes might be the trigger for causing ALS pathomechanisms but a single gene with direct metabolic disturbance has not been found or linked yet [26].

### 3.6. Friedreich Ataxia

This is the most widely observed hereditary ataxia in humans. It occurs in a person due to homozygous or compound heterozygous transformations in the FXN gene of a human being [27]. Some of the symptoms of this disease are dysarthria, areflexia, and gait ataxia [28]. The leading cause of death in Friedrich ataxia is cardiomyopathy [29]. It is a rare disease that occurs due to a deficiency of a mitochondrial matrix protein called frataxin. This affects about 1 out of 50,000 individuals worldwide [30].

### 3.7. Spinal Muscular Atrophy

Spinal muscular atrophy (SMA) is the cause of the highest number of deaths in children and is of genetic origin. It is a neuromuscular disorder that makes a person unable to sit, stand, or walk [31]. The symptoms of this disease are a weakening of skeletal and respiratory muscles over time and the degeneration of lower motor neurons in humans [32]. Some research showed that gene therapy was effective in children under the age of 6 months for treating SMA [33]. The most common and dangerous type of SMA observed in humans is type 1 SMA [34].

## 4. Computational Intelligence Techniques for Diagnosing Degenerative Nerve Diseases

### 4.1. Machine Learning Techniques

**Figure 4:**
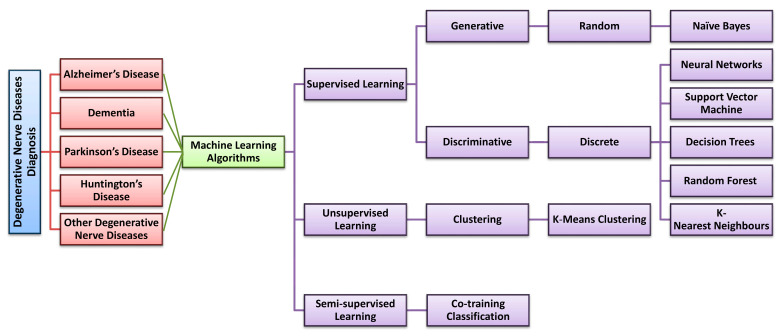
Machine learning models used for degenerative nerve disease diagnosis in this review: a tree illustration.

In this section, we describe several supervised, unsupervised, and semi-supervised models and their potential applications in the diagnosis of degenerative nerve diseases in humans. Data that were used and the methodology employed are described along with the limitations of each model [35,36,37,38,39,40,41,42,43,44,45,46,47,48,49,50,51,52,53,54,55,56,57,58,59,60,61,62,63,64,65,66,67,68,69,70,71,72,73,74,75]. Figure 4 represents machine learning models for degenerative nerve disease diagnosis used in this review through a tree illustration. 

#### 4.1.1. Artificial Neural Network

Artificial neural networks are models that are used to simulate the human nervous system. They have several layers of “neurons” or nodes, depending on the computations required in the problem statement.

In the field of degenerative nerve disease diagnosis, artificial neural networks are used to differentiate the dataset containing medical records of patients having Alzheimer’s disease from those patients who have mild cognitive impairment, only using unprocessed data from their electroencephalogram [35]. This aids medical practitioners in accelerating the diagnosis for such patients. Recently, a study aiming to identify the use case of ANNs for the diagnosis of degenerative disorders managed to obtain a sensitivity of 93.8% using the ANN algorithm on the brain SPECT (single photon emission computerized tomography) of the patients’ records. This successfully proved the use case of ANNs in clinical practice, but after comparing it with discriminatory analysis, which also achieved a sensitivity of 86.1%, there is no significant benefit of using an ANN algorithm over other algorithms [36]. This limitation may be overcome by supplying the dataset with more data, which would aid in increasing the accuracies of these neural network models. Additionally, by employing many biomarker modalities, the accuracy may improve in identifying mild cognitive impairment in the patients [37].

ANN is also making strides in the field of diagnosing dementia. A study conducted recently using real world data of patients from a hospital in Brazil constructed an algorithm using C++ in which a regression model subject to feedforward ANN was used to classify different stages that the individuals went through in the spread of the disease in their body. The algorithm was trained through 200,000 iterations and had three elements to its output layers. One element showed individuals with dementia, one showed individuals with MCI, and the last element showed non-symptomatic people [38].

#### 4.1.2. Support Vector Machine

In SVMs, the object that we want to classify is represented as a point in the n-dimensional space, and the coordinates of this point are known as features. SVMs perform the classification test by drawing a hyperplane to differentiate the points [39].

SVMs can be of great importance to classify patients with AD. This was showcased by a study on Alzheimer’s disease in Thailand, in which subjects were randomly assigned to training and testing groups for the validation and construction of an SVM model. The study showed that clinical parameters, used with SVM classifiers, showed high accuracy when diagnosing patients with Alzheimer’s [40]. However, this study was based on a small group of subjects, including patients who were clinically diagnosed with dementia along with those who came from a community survey. Therefore, due to the smaller scale and lack of diversity, the generalization of the study might produce a different accuracy. Another recent study used the dragonfly technique (DA), a meta-heuristic algorithm, to improve the input data for a better performance of the SVM. Unlike particle swarm optimization, artificial bee colony and ant colony optimization, and other popular meta-heuristic algorithms that have only one target, DA is special in that it has both a target and an enemy, i.e., in DA, flies will always be between the target (upper bound) and the enemy (lower bound) [41]. This helps in the selection of optimal parameters, thus aiding the SVM model and showing a significant increase in accuracy.

A key limitation observed when using SVMs for the classification of dementia and other diseases is that, in the datasets being used, if the classification and normalization of the subject groups are not accurate, then this might sabotage the estimation of dementia in elderly people. Additionally, a large amount of test subjects is needed to form an enriched dataset to increase accuracies of the various models being used [42].

SVM also has applications in the diagnosis of Parkinson’s disease. In a recent study, the authors used a dataset consisting of human voice patterns to find out if individuals suffer from Parkinson’s or remain asymptomatic. A non-linear SVM was used to test all 22 voice patterns in the dataset by applying a ten-fold cross-validation in the training and testing stages of the model. It achieved a good accuracy of 92.13% and was fruitful in differentiating between the individuals based on their voice patterns [43].

#### 4.1.3. K-Means Clustering

The goal of the k-means algorithm is to group similar data points together to form predefined groups of clusters. Two data points that are closer together are more similar compared to two data points that are farther apart [44].

One of the most noteworthy methods used to detect Parkinson’s in a patient is by checking if he/she exhibits freezing of gait (FOG) in their motor behavior. FOG is the behavior observed in a Parkinson’s patient wherein they have a lot of distress in demonstrating their motor skills. In the process of detecting this, the dataset is clustered into several mini batches so that the training time may reduce and increase efficiency. The value for k is set equal to 2 and is given a set of entropy, the clusters thus formed are given to the FOG detection system in a random order, and the results are observed [45]. However, this system suffers from a lower sampling frequency, which reduces the amount of data that could be updated and potentially affects the performance of the current model. Another development in the application of k-means clustering in the field of neuro-biology was deployed in the detection of Alzheimer’s disease. The MRI images are obtained from the dataset, in which top-hat and bottom-hat filtering is used to increase the image quality. The filtered images are then processed using k-means to obtain the results [46]. An early diagnosis of the diseases might also be aided by studying the functional connectivity of patients. A study for patients suffering from AD and dementia with Lewy bodies using k-mean clustering and dynamic analyses showed the differences in the functional connectivity of the patients and the healthy population, but it did not show any significant difference in patients with a different severity of the diseases. Further enhancements to the usage of k-means clustering are necessary to overcome issues such as the misdiagnosis of diseases. For example, the models could not find the difference between patients suffering from Lewy body dementia (LBD) who took dopaminergic medicines and patients who did not [47].

K-means clustering also has potential applications in diagnosing Huntington’s disease. Though this was only tested on mice, the algorithm successfully classified the subjects into clusters and gave the differentiation between mice who might suffer from Huntington’s and mice who did not show any symptoms. The model was trained using original data from 30 mice and was programmed on MATLAB software. They tested their k-means algorithm in both an ROC supervised and unsupervised manner to find out the best yield of results. The ROC supervised model came out as the better method and was correct in classifying all the mice in their respective brackets of disease probability [48].

#### 4.1.4. Decision Tree

A decision tree splits the dataset recursively using decision nodes. It is a type of supervised learning algorithm and is mainly used for classification problems. A decision tree has three aspects to it: internal nodes, branches and leaf nodes [49].

In a recent study, a heterogeneous model comprising decision tree, rule induction, random forest, and a generalized linear model was used for Huntington’s disease diagnosis. The model was trained using a cross-validation strategy [50]. It identified the genes that are found in a person with Huntington’s disease. A limitation of the model is that the mutant HTT gene may interfere with the promotion of Huntington’s disease pathogenesis.

Decision trees can also be used in the early detection of Alzheimer’s disease in people. One study outlined the decision tree model in such a way that it was optimized using entropy and information gain [51]. This is a novel approach and showed significant results in diagnosing the diseases at an early stage. Another study used decision trees in compliance with hyper parameter tuning (HPT) for the early diagnosis of AD. The study compared various algorithms on different metrics. The decision tree showed a high precision percentage but a low recall percentage. It also showed a much lower accuracy percentage compared to SVM and random forest when the OASIS dataset was used [52]. In a hybrid model, decision trees along with Bayesian belief networks and Naïve Bayes classification algorithms were used as a data mining transcript for the diagnosis of dementia in humans. This transcript mined words from the handwriting dataset of 605 medical records to tell whether the individual might potentially suffer from dementia in the near future or is asymptomatic and safe [53].

#### 4.1.5. Random Forest

This algorithm creates a ‘forest’ with several decision ‘trees’. In general, more trees in the forest lead to more robust predictions, thus giving us a higher accuracy. This method is the same as constructing a decision in a decision tree [54].

In the detection of Parkinson’s disease, the random forest algorithm was combined with PCA (principal component analysis), in which the training set was utilized to make six prediction models for Parkinson’s disease based on different handwritten exams that the patients were asked to write [55]. This was followed by a plural voting of all decision trees, thus giving us the final classification result. A limitation of the model is that no general feature extraction method was given for different handwritings to improve the final voting performance. In recent times, bootstrapping was performed, in which a random sample is taken from the sample with replacement of data [56]. This allows the algorithm to choose from a huge variety of trees of different sizes and shapes to obtain better results. However, the replacement of data can cause overfitting. Another model that aims to provide the early diagnosis of Parkinson’s disease uses explanatory variables that are randomly chosen from the samples. The study showed that random forests show better prediction accuracy than regression models or decision trees and also bagging models when there are many input variables. However, the study failed to take into account the obsessive-compulsive symptoms common in patients with PD and also did not include biomarkers such as CFS. To fetch better results and higher accuracies, a weighted voting system can be utilized in random forest algorithms [57]. Random forest also has applications in the diagnosis of Alzheimer’s disease in humans. In one research article, the authors proposed a method through which they identified specific proteins in the human body that are associated with AD in humans. They used a hybrid method of random forest with logistic regression that randomly extracted 40 features from the dataset, and then the AD-related proteins were found [58].

#### 4.1.6. Naïve Bayes

Naïve Bayes is a machine learning algorithm based on the Bayes theorem of conditional probability. It assumes that the presence of a feature in a class is unrelated to the presence of any other feature, even if these features depend on each other [59].

Naïve Bayes has great potential in aiding disease diagnosis. Class imbalance, a major problem observed in large datasets, can potentially be resolved using the Naïve Bayes algorithm, as it multiplied the class prior probability with the likelihood of the disease in the datasets of patients suffering from dementia and gave promising results [60]. However, the model may show a selection bias that might cause the misdiagnosis of diseases if implemented in a clinical setting. In another study for the detection of AD, processed MRI features were taken in partition vectors and fed to the Naïve Bayes model [61]. This made the training of the model much smoother and more efficient in execution time, but the model failed to incorporate data from blood cell content, protein–protein and gene–gene interactions, etc. A general limitation in Parkinson’s disease diagnosis when using Naïve Bayes is that it may have some fluctuations that affect the result of the final output [62].

#### 4.1.7. K-Nearest Neighbor (KNN)

This algorithm scans for all past experiences and finds the k closest experiences; these data points or experiences are the k-nearest neighbors. It is based on supervised learning that assumes similarity between the new case and the available cases [63].

KNN can also be used in Alzheimer’s diagnosis in humans. A combination of KNN along with deep neural networks can be used with a probability combination to show the representative accuracy when 3D MRI scans are fed as data to the model [64]. Additionally, KNN can be used to detect dementia in subjects where EEG signals are fed to it as inputs [65]. However, the current research focus has shifted to incorporate linguistic features to feed the model so that the results obtained are more accurate and faster for dementia detection [66].

#### 4.1.8. Extreme Learning Machine

Extreme machine learning (ELM) models are used for classification, regression, clustering, and compression with a single layer or multiple layers of hidden nodes [67].

It has large applications in the diagnosis of Alzheimer’s disease. One such application classifies the gait pattern using ELM-based models [68]. They have also been used to identify and separate those diagnosed with mild cognitive impairment from later stages of Alzheimer’s disease. This was conducted to tackle the increasing number of Alzheimer’s patients so that those suffering from severe Alzheimer’s can be given care separately [69].

ELM models are also used to diagnose and classify patients with Parkinson’s disorder. New frameworks to improve accuracy and scalability are researched regularly. One such method optimized the extreme learning model using the bat algorithm; this produced a significant rise in accuracy [70]. Another method used local binary pattern descriptors to classify patients with Parkinson’s disorder based on a spectrogram [71]. However, both these models were not tested on databases with large-scale images; hence, their accuracy might vary.

Huntington’s disease (HD), another degenerative nerve disease, is plagued by missing data in its datasets due to the rarity of the disease. This causes issues with accuracy in the diagnosis of the disease. To solve this problem, ELM models can be used. One way is by using brute force, where the missing values are imputed using previous observations. This gives better results than just ignoring the missing values [72]. However, the limitation lies in that the assumptions might vary as the heterogeneity increases, which causes a decrease in the quality of prediction as larger time intervals are considered. Another method uses multiple imputations with ELM to fill the missing value. Through this method, the prediction of Huntington’s disease is possible through MRI scans, as early as 10 years prior to the onset of the disease [73].

#### 4.1.9. Co-Training Classification

Co-training classification is a type of proxy label method under SSL (semi-supervised learning). These models take advantage of a model trained on a given labeled set to create more training examples by labeling instances of an unlabeled set based on certain heuristics [74]. In the detection of Alzheimer’s diseases in humans, this SSL method can be used by employing MRI and PET (positron emission tomography) images as data, and MCI (mild cognitive impairment) data were used as the unlabeled sample [75]. This method is promising for the classification of AD but also needs to be used with other co-training algorithms to improve the general performance of the framework.

### 4.2. Limitations: Machine Learning Techniques

The limitation of using machine learning for the diagnosis of degenerative nerve diseases is mainly the unavailability of data. This in turn translates into many other hindrances such as the exclusion of biomarkers in the datasets for diseases such as Alzheimer’s. In the case of SVMs specifically, large kernel cases are a big hindrance to the data being processed, which results in lower sensitivity.

### 4.3. Inferences-Machine Learning Techniques

In all the machine learning algorithms surveyed, we found out that ANN brags of a consistent record of maintaining accuracy, sensitivity, and specificity of almost 98% and above. This is observed in cases where the model is pre-trained and not. The reason for such high metrics is a combination of how ANNs can learn and model non-linear relationships between the input and the output along with the increase in availability of much more complex and enriched datasets in this field. Table 1 presents a summary of works on machine learning for degenerative nerve disease diagnosis.

### 4.4. Deep Learning Models

**Figure 5:**
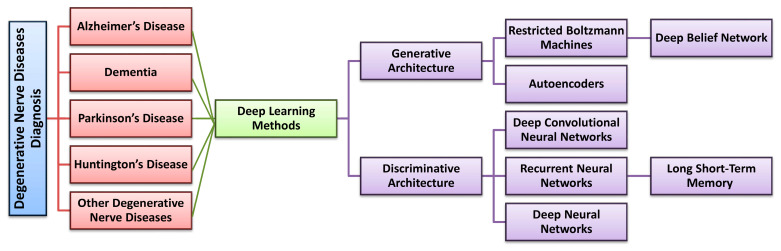
Deep learning models used for degenerative nerve disease diagnosis in this review as a tree illustration.

In this section, we describe several supervised, unsupervised, and semi-supervised deep learning models and their potential applications in the diagnosis of degenerative nerve diseases in humans. Data that were used and the methodology employed are described along with the limitations of each model [76,77,78,79,80,81,82,83,84,85,86,87,88,89,90,91,92,93,94,95,96,97,98,99,100,101,102,103,104,105,106]. Figure 5 represents the deep learning models for degenerative nerve disease diagnosis used in this review through a tree illustration. 

#### 4.4.1. Recurrent Neural Networks and Long Short-Term Memory

Recurrent neural networks or RNNs are connectionist models capable of processing sequential data with varying lengths and representing time dependencies. Their ability to capture sequences via cycles in nodes and long short-term memory (LSTM) and bidirectional (BRNN) architectures have made RNNs useful for the diagnosis of various degenerative nerve diseases, such as Alzheimer’s disease, dementia, and others [76]. To conduct this, RNNs generally use classification, regression, and clustering performed by extracting information from sequences of data [77]. Additionally, by applying LSTM to a model, the algorithm can learn time dependencies and control the exposure of memory content [78].

One of the major applications of RNNs is to identify symptoms of Alzheimer’s disease and dementia at an early stage and predict and track the progression of the disorders, even with irregular time intervals by accounting for longitudinal temporal patterns [79]. However, this model is only useful for predicting disease progression at later stages and cannot be applied for an early diagnosis of the disease. Another application of RNN is in smart homes for the human activity recognition of people with degenerative nerve diseases, thus reducing the need for very frequent hospital visits [80]. However, this method of diagnosis is questionable due to a possible breach of the patient’s privacy.

Other challenges in the implementation of RNN models are a lack of available datasets and missing information in the existing datasets that needs to be filled out before the processing. A novel method to overcome these challenges is using a framework that can adaptively impute missing values and predict the future progression of the disease from a subject’s previously available data [81]. Another medium to overcome these challenges while avoiding this pre-processing step is the use of an LSTM algorithm that, instead of imputing the missing values, tackles the incomplete data using a generalized formulation of backpropagation through time. This algorithm is also used to model temporal dependencies among measurements in the ADNI data via sequence-to-sequence learning [82]. This training method outperforms models that rely on the imputation of missing data before standard LSTM network training.

#### 4.4.2. Autoencoder

Autoencoders are unsupervised artificial neural networks that are used to reduce data dimensions by ignoring the noise in data [83]. They have wide applications in healthcare and are useful in diagnosing degenerative nerve diseases such as Parkinson’s disease, Alzheimer’s disease, and amyotrophic lateral sclerosis.

Autoencoders, when applied with normative models for understanding the biology fundamental to degenerative nerve diseases such as AD at the level of an individual, are useful for quantifying deviations in regional brain volumes of patients with AD compared to healthy patients. These deviations also reflect the severity of the disease and its progression [84]. A modeling approach, termed “NormVAE”, was successful in identifying and analyzing the brain regions associated with the patient-level deviations and also produced deviation maps more sensitive to disease staging within AD using variational autoencoders to estimate patient-level deviations with uncertainty estimates [85]. One of the limitations of this approach is that the dataset used for the model consists of patients only from a single country, which cannot be generalized for the global population. Thus, using it on a global scale might lead to false positives and inappropriate treatment. Variational autoencoders are also used for producing future brain F-Fluorodeoxyglucose PET images, which are used to predict future brain topography and give insights into possible degenerative nerve diseases [86].

In the early diagnosis of Parkinson’s disease, another application of autoencoders was based on vocal impairments at an early stage. These vocal impairments can be identified using sparse autoencoders or stacked autoencoders, which can then identify the subjects who are patients of PD [87,88]. This is a novel approach compared to MRIs and CT and has shown great results in identifying patients in the early stages of PD. It is however limited due to a shortage of rich data. Autoencoders can also be used for the diagnosis of amyotrophic lateral sclerosis, for which the raw data are denoised using stacked autoencoders, which are then used to predict the disease [89]. This approach has shown high accuracy and promising results and may very well become a crucial method for the early diagnosis of ASL in the future.

#### 4.4.3. Deep Belief Network

A deep belief network or DBN is a generative graphical model containing multiple layers of variables that are connected with each other. It can be used to solve unsupervised as well as supervised learning tasks [90].

There has been rapid development in the utilization of DBN-based models, especially in healthcare. DBN models are used to diagnose Alzheimer’s, Parkinson’s, etc. Using the DBN model for the classification of MRI slices is an efficient method for the diagnosis of Alzheimer’s disease. This has been performed using various other models such as ANN, SVM, and LDA, but the DBN-based model showed much higher accuracy and more promising results [91]. Recently, it was identified that protein expression data can also be used as a risk marker for Alzheimer’s disease; however, traditional models based on other algorithms are unable to make full use of these data. Here, DBN-based models show much better results by identifying proteomic risk markers and reinforcing the link between metabolic risk factors and Alzheimer’s disease. They also provide evidence that adiponectin-linked pathways could be a therapeutic drug target. However, the limitation in this model is that there is no general and intuitive way to visualize training weights, which makes implementation difficult [92,93]. Hence, it is not yet implementable for clinical use.

DBN models can also be used for the diagnosis of Parkinson’s disease and can implemented using the self-organizing map clustering approach with the aid of support vector regression to improve the accuracy and scalability of prediction [94]. However, the current literature is focused on using ELM as a supervised learning technique, which affects the scalability of the model. Using alternatives such as ensemble learning techniques is more beneficial to improve the use case of the model. Along with this, the dataset used for the model had a limited number of features; hence, a change in accuracy can be expected with different datasets.

#### 4.4.4. Deep Convolutional Neural Network

In deep learning, a convolutional neural network (CNN) is a class of artificial neural networks commonly applied to analyze visual imagery. CNNs are regularized fully connected networks that take advantage of the hierarchical pattern in data and assemble patterns of increasing complexity using smaller and simpler patterns enchased in their filters [95,96].

CNNs are very valuable in the diagnosis of Alzheimer’s disease using MRI, as they can perform binary classifications to identify irregularities with high precision and aid in the early diagnosis of patients; this has further been improved using the novel framework of a 12-layered CNN [97]. However, this model does not support multiclass classification, which is important for classifying the severity of the disorder. An alternative method pre-processes structural MRls in a strict pipeline, and instead of parcellating regions of interest, each volume is re-sliced and put into a DCNN directly [98]. Another way is to use multi-class classifications for brain MRI data analysis to identify different stages of Alzheimer’s disease and contribute to its early diagnosis [99].

CNNs are also useful in the diagnosis of Parkinson’s disease, which can be identified by classifying speech signals with the help of a CNN architecture for one-dimensional signal processing [100] and handwriting, which can be classified using various features that are learned from one’s handwriting that are then extracted by CNNs [101]. The limitation of this model is its slow speed of operations and large training time.

Recently, CNNs have also been utilized for the identification of amyotrophic lateral sclerosis (ALS) risk variants in noncoding regions. This is a significant step as the identification of these risk variants has been difficult and a major issue in the diagnosis of ALS. To achieve this, a one-dimensional convolution with a vector is performed that is compatible with the linear DNA strands [102]. However, the model relies on the assumption that the locus contains at least one casual variant; hence, the presence of false-positive GWAS signals may lead to a significant loss in performance.

#### 4.4.5. Deep Neural Network

Deep neural networks or DNNs are complex multilayered neural networks that are used for convoluted calculations and classification. They have promising applications in solving high complexity problems such as the diagnosis of degenerative disorders [103]. A recent study comparing the performance of DNNs with various ML models for the diagnosis of Parkinson’s disease, using digital bio-markers and speech records as datasets, favored the use of DNNs. It used an open-source tool, OpenSmile, to extract two feature sets that were used as inputs for the three-layered DNN. The results showed an 85% accuracy, outperforming the average clinical diagnosis accuracy of non-experts. The paper provided a good look at the potential of DNNs for disease diagnosis; however, it only utilized a single biomarker. An increase in biomarkers should increase the accuracy of DNNs, but it might also propel some other algorithm above the DNN [104]. Obtaining large-scale datasets is a difficult task, and smaller datasets limit the accuracy of a model. To overcome this, a novel method used transfer learning with a deep neural network to produce a robust model that can work well on smaller datasets. This model is highly suitable for the study of less-known degenerative diseases, which usually have small datasets. However, this method is not a replacement of the required data collection that is essential for the study of these diseases [105].

DNNs are also well suited for identifying complex relations between the genotype and phenotype of an individual. These complex relations might be the key to solving degenerative diseases such as ALS, whose genetic basis is still not completely understood. Recent research focusing on the use of DNNs for identifying the ALS-associated regions using genome data as input showed promising results with an accuracy of 77%. The study only considered the genomic information of four chromosomes and applied a two-step approach using a CNN model followed by ALS-Net, whose architecture is based on the structure of genome data. This article gave significant insights in the diagnosis of ALS and the relations between ALS and genotypes. However, the article is limited by the lower number of chromosomes included. Including other chromosomes might be a challenging task; yet if it is overcome, it will significantly improve the model’s accuracy [106].

### 4.5. Limitations-Deep Learning Models

Various deep learning models mentioned above have shown promising results and the potential for playing a role in degenerative nerve disease diagnosis; yet, these models are plagued by a number of limitations that need to be overcome. The most prominent limitation is a lack of suitable datasets. Similar to machine learning models, deep learning models also suffer in accuracy and performance because of low-quality datasets with missing values and a smaller amount of data. Another limitation faced by a number of deep learning models is the slow speed of operations. The models also suffer from adversarial noise, which could be a big issue for practical clinical use, since most models are not robust enough to be scaled to a clinical level. Lastly, another major limitation common in both ML and DL models is the privacy protection of patient data, which has received surprisingly low attention from any research article.

### 4.6. Inferences-Deep Learning Models

While all the DL algorithms surveyed have shown promising results, deep neural networks, including deep CNN and deep belief networks, have shown tremendous potential. The models based on these algorithms have shown much better results than even their machine learning counterparts. These models have shown accuracies as high as 97%, and models based on them have also provided good imputation methods for missing values in medical datasets. The capacity of these algorithms to solve high complexity problems is greater than other algorithms discussed in the paper, which gives them an edge in real-life application for degenerative nerve disease diagnosis. Table 2 presents a summary of works on deep learning for degenerative nerve disease diagnosis.

### 4.7. Other Computational Intelligence Models

While the algorithms described in the above subsections of machine learning and deep learning have been prevalent in recent research for the diagnosis of degenerative nerve disorders, several other algorithms were also tried to create new robust models and overcome the current limitations. Some of these are described in the following sections.

#### 4.7.1. Probabilistic Neural Network

These are used for classification and pattern recognition and allow the compact description of complex relationships between random variables [107]. This makes it useful for applications in disease diagnosis. A research article suggested a model where PNN was optimized using particle swarm optimization (PSO) for disease prediction. This method can have useful results for the diagnosis of disorders such as Alzheimer’s disease [108]. Another useful application of PNN could be in the diagnosis of Parkinson’s disease. An enhanced PNN or EPNN, which was developed by modifying the Gaussian kernel based on the diversity of the training dataset [109], showed significant potential when used on the Parkinson’s Progression Markers Initiative dataset to classify patients of Parkinson’s disease [110].

#### 4.7.2. Deep Residual Network

These have useful applications in degenerative nerve disorder diagnosis, as they enable the construction of very deep networks without degradation in performance. A research article used a residual network-based model for classifying AD. The ResNet model used in the article predicted AD in six stages with an accuracy of 97%, which is comparable to state-of-the-art results. The study provided solid grounds to use ResNet for AD progression as well as other applications such as drug discovery [111]. Another article discussed the application of residual networks in the diagnosis of Parkinson’s disease. The article used a 3D residual CNN model to extract invisible features and improve accuracy as well as discover saliency features in critical regions of the brain. The results of the article were promising, with great diagnosis accuracy; however, the dataset used was not enough for identifying clinical use as of now [112].

## 5. Open Challenges-Degenerative Nerve Diseases Diagnosis

**Figure 6:**
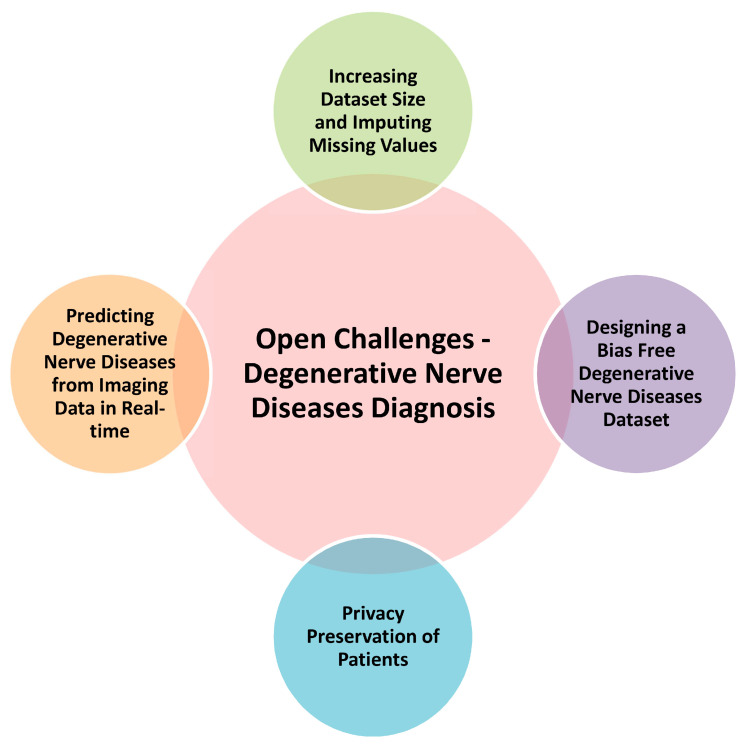
Open challenges in degenerative nerve disease diagnosis.

As discussed above, although ML and DL algorithms have shown high potential for application in degenerative nerve disease diagnosis, they are still plagued by a large number of limitations. Some of these limitations are common to both ML and DL algorithms, while others are more specific. Through these limitations, we can infer a set of open challenges that need to be overcome to practically use the above-mentioned models on a larger scale at a clinical level. Figure 6 represents the open challenges in degenerative nerve diseases diagnosis.

Current open challenges include those listed in the following sections.

### 5.1. Increasing Dataset Size and Imputing Missing Values

One of the biggest problems faced by almost every model is a small or partial dataset. This problem is worse for rarer diseases, such as Huntington’s disease or ALS, that often suffer from a lack of large and suitable datasets. This further leads to less robust models since the models suffer from a lack of appropriate data for the different stages of the diseases. Imputing missing values is another major challenge that is faced even while working with datasets of more common diseases, such as Parkinson’s or AD. Imputing correct values is tricky, as careless imputation can lead to incorrect conclusions. Thus, enhancing the available datasets of less-common diseases and further developing the methods of data imputation is critical to produce robust models that can be applied at the grassroots level.

### 5.2. Designing a Bias Free Degenerative Nerve Diseases Dataset

Biases in degenerative nerve disorder datasets are a common issue. A biased dataset can significantly affect the accuracy and general performance of a model. Any decrease in model performance impacts its usability. A lower accuracy model can cause misdiagnosis, which could be catastrophic for a patient. Creating bias-free datasets is a significant step towards improving ML and DL models alike and can take us another step closer to the actual implementation of these models for an early diagnosis of degenerative nerve disorders.

### 5.3. Privacy Preservation of Patients

As a major concern in all aspects of society, maintaining patient privacy is very crucial for any computer assistant system. Patient privacy is often ignored when talking about a new model or system for disease diagnosis, yet it is just as important as a model’s accuracy. Not maintaining high privacy standards and data leakage can lead to unnecessary problems in a patient’s life, leaving them vulnerable. Various suggested models that propose the daily tracking of patient activities do not mention how they plan to preserve the privacy of their users. The protection of these data is a critical requirement, and appropriate steps need to be taken to overcome this challenge; otherwise, even the models with the highest performance cannot be used in a real-world clinical settings.

### 5.4. Predicting Degenerative Nerve Diseases from Imaging Data in Real-Time

A major limitation of the discussed models is the slow speed of operations. This reduces the applicability of the prediction models in real-life clinical use. Overcoming this challenge will enable the real-time diagnosis of patients, which will allow doctors to take necessary steps at an early stage, and this can significantly lower the disease progression speed.

## 6. Future Research Directions: Degenerative Nerve Diseases Diagnosis

**Figure 7:**
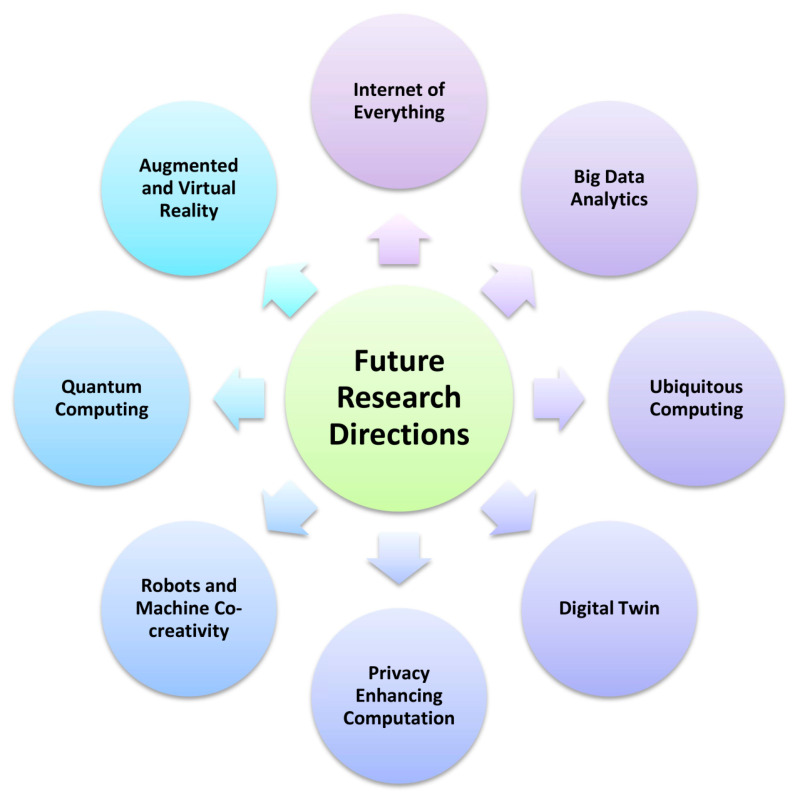
Future research directions in degenerative nerve disease diagnosis.

Similar to machine learning and deep learning algorithms, many other technologies have potential for aiding degenerative nerve disorder diagnosis. These methods could also be used to circumspect some of the open challenges we face while working with machine learning models. Some of these are listed below. Figure 7 illustrates the future research directions in degenerative nerve diseases diagnosis.

### 6.1. Internet of Everything

IoT consists of devices or “things” that are connected through various sensors. IoT devices, through mobile phones, have become an inseparable part of our lives. This has made them an alternative solution with huge potential in the early diagnosis of degenerative nerve diseases. Wearable technologies, such as smartwatches, can monitor and record our daily schedule in a systematic form, and this data can be used to identify irregularities in the behavior of a person and identify if they have a degenerative nerve disease [113]. An example of this is the diagnosis of Parkinson’s disease by monitoring various features of the patient such as gait, sleep disorders, etc. This method is much cheaper when compared to various others and has vast potential, as the collected data can be directly used to verify the patient’s manually uploaded data and can also be used for identifying these disorders and linking them with various degenerative nerve diseases, early and unsupervised [114].

### 6.2. Ubiquitous Computing

Ubiquitous computing, or pervasive computing, is the presence of computational power all around us. This is a growing field and may very well be partially achieved by the next decade. A person suffering from a degenerative nerve disease can be identified very quickly in a ubiquitous computing environment, as all their habits and lifestyle routines are recorded in the machine’s database, and subtle irregularities can also be identified efficiently [115]. An example of this is a mobile app, STOP [116], which was made to detect and monitor patients with Parkinson’s disease. The app uses a ball game to monitor the motor skills of a patient, a medication journal for users to log their daily medications, and a daily survey to assess the severity of the disorder. This app, although tested on a small number of patients, produced good results. It shows that, as processes are automated further through ubiquitous computing, the diagnosis and monitoring of degenerative nerve diseases are bound to become more convenient.

### 6.3. Augmented Reality (AR) and Virtual Reality (VR)

Virtual reality (VR) is a computer-generated world that mimics real life. In the last decade, we have seen a significant increase in research aimed at using virtual reality and augmented reality for healthcare. One such example is an eye-tracked VR for the remote diagnosis of degenerative nerve diseases, in which ocular irregularities can be identified and evoked. This makes it possible for patients to be tested for degenerative nerve diseases even at relatively smaller institutions and eliminates travel issues that might have caused patients to postpone going for diagnosis [117]. Another use of AR could be in mapping and visualizing the brains of patients suffering from degenerative nerve diseases such as Alzheimer’s disease. This works by visualizing any abnormal clumps present in the brain. Using AR this way allows a much better analysis of the brain and evaluation of the diseases [118]. Apart from the diagnosis, another application of VR could be in improving the cognitive functions of a patient with a degenerative nerve disease such as dementia [119]; however, there is no substantial proof of this, and hence, this application is still debatable and is subject to change as further research continues.

### 6.4. Robots and Machine Co-Creativity

Machine co-creativity is the use of machines and AI to create original artistic content such as music and paintings. It is a very futuristic technology and has not been properly developed yet. Machine co-creativity has applications for the diagnosis of degenerative nerve diseases such as dementia to assess and understand the stage in which the patient tracks the progress of the disorder and can also be used as a leisure activity by the patients [120]. An example of this is music. Research has shown that musical memory is usually retained by dementia patients. Here, machine co-creativity can be used to help the patient generate music, which can be used to understand the stage of the patient’s dementia and also allows the patient to enjoy a leisure activity [121].

### 6.5. Big Data Analytics

Data analytics are the new way to deal with the ever-growing amount of data available to us. Traditional methods are not able to compute and handle the terabytes of data out there. Moving to Big data can greatly aid in the diagnosis of degenerative nerve diseases as the amount of data available for research will increase by a significant amount, overcoming the difficulty of the lack of data faced by several machine learning and deep learning models [122]. Additionally, in the field of neurobiology where a lot of data from previous patients and the data of current subjects are available, the method to handle such heavy datasets becomes a lot more efficient when Big data is employed. This can even handle image data such as brain MRI scans [123].

Apart from diagnosis, data analytics are also useful for managing patients. Data mining, a subset of data analytics, can also be a great tool for identifying patterns and relations between degenerative nerve diseases and other factors. This can be helpful in the early diagnosis of these disorders and in managing the existing patients suffering from them. An example could be a study to determine the relations between dementia and other kinds of illnesses [124], which can help in managing the patients accordingly by identifying their risk of other diseases based on their age, gender, etc.

### 6.6. Quantum Computing

As one of the most up and coming fields, quantum computing is used to solve complex problems and has vast applications in AI. The technology has vast potential and can play a significant role in the diagnosis of degenerative nerve diseases in the future. An example of this would be using quantum computing with Naïve Bayes, KNN, decision trees and artificial neural networks to predict Parkinson’s disease [125]. However, there is still a lack of work using quantum computing for the diagnosis of degenerative nerve diseases. Hopefully, this can be overcome in the future as more attention is given to research on quantum computing.

### 6.7. Digital Twin

Digital twin is the virtual model of a physical product or process that is identical to its physical counterpart. The technology is being developed for use in various industries to aid in the manufacturing of different products. Recently, it was suggested that digital twinning can also be used for creating human virtual twins, and if successful, this can help diagnose various health problems much earlier in life. These problems include degenerative nerve diseases such as multiple sclerosis and Alzheimer’s disease.

Using a conditional restricted Boltzmann machine, ‘digital subjects’ can be created, and these subjects are statistically identical to the patients and can play a major role in mapping the progression of multiple sclerosis. However, it is difficult to do so because the disease progression varies and depends on many components. This limitation can be overcome by including additional data that can help to create a more accurate digital subject and reduce the complexity of endpoint measures and multidimensional courses of the disease [126].

### 6.8. Privacy Enhancing Computation

One of the biggest challenges in implementing ML and DL in the diagnosis of degenerative nerve diseases is the lack of available datasets. Privacy concerns are a major reason behind this, as it is difficult and risky to obtain data from individuals if the privacy of their data cannot be guaranteed. Recent developments in privacy-enhancing computation, although in the early stages, look very promising. Two popular techniques that are being developed to enhance user privacy are homomorphic encryption and secure multiparty computation. Homomorphic encryption allows direct computation on encrypted data, and this means that the data can be freely shared in an encrypted state and the organization requesting the data can perform the computation without needing to decrypt it. However, this method is expensive and still under development. Secure multiparty computation protocols are made to protect the privacy of data shared between multiple parties working together so that the data being shared by each of them are protected from the outside as well as the other parties working with whom the data need to be shared. However, the adoption of these protocols can be difficult and might also affect the flexibility of work [127].

Multiparty homomorphic encryption is a combination of homomorphic encryption and secure multiparty computation, which was designed to bring down the overhead costs and make privacy enhancement more practical. It is used to perform the most efficient approach in a given workflow [128]. Although it is still being developed to be more effective, multiparty homomorphic encryption can be useful for privacy enhancement, which allows the sharing of data while protecting privacy, increasing the amount of data available for further research on various medical conditions, including the diagnosis of degenerative nerve diseases.

## 7. Conclusions

Degenerative nerve diseases have been a popular topic of interest for a very long time. These disorders are untreatable and worsen the patient’s condition with time. The only measure we can currently take is to slow down the progression of these diseases. The early diagnosis of these diseases can enable patients to practice preventive measures before the disease progresses to an uncontrollable stage; hence, the early diagnosis and progression tracking of these disorders are crucial. Through this paper, we assessed the role of machine learning and deep learning in the diagnosis of these disorders and identified various algorithms that have shown promising results when used for the diagnosis of degenerative nerve diseases. Recent developments in each of these algorithms, such as the use of a hybrid clustering and DBN to improve the scalability and accuracy of classification of patients with Parkinson’s disease or a combination of sliding window approach and k-means clustering and dynamic network analyses for diagnosing dementia patients, show the immense potential that the various ML and DL algorithms have in the early diagnosis and tracking of these diseases. In the models we surveyed, we found that artificial neural networks and deep neural networks, including deep CNNs and deep belief networks, are the most promising algorithms for detecting degenerative nerve diseases. However, we found out that the use of ML and DL is still plagued by major challenges such as a lack of available data and lower scalability and accuracy. Hence, the future direction of research on using ML and DL to diagnose degenerative nerve diseases should be to overcome these challenges. Apart from machine learning and deep learning, there are a few other promising technologies such as the Internet of Things, digital twin, quantum computing, and Big data analytics, among others, that can potentially be helpful in the diagnosis of degenerative nerve diseases in the future.

## Figures and Tables

**Table 1:** A summary of works on machine learning for degenerative nerve disease diagnosis.

Ref	Degenerative Nerve Diseases	Machine Learning Approaches Used	Learning Model	Dataset Used	Pre-Trained or Not	Key Contribution	Limitations	Metrics
[36]	Alzheimer’s disease	Artificial neural network	Supervised learning	Collected their own private dataset	Not pre-trained	Uses brain SPECT records of the patient to get a much more accurate result.	No significant difference in the sensitivity in comparison with discriminatory analysis.	Sensitivity: 93.8% Specificity: 100%
[37]	Alzheimer’s disease	Neural network	Supervised learning	ADNI dataset ADNI + Milan dataset	Pre-trained	A single cross section of a brain MRI scan is fed to the model to predict whether the subject has Alzheimer’s or not.	The model was not tested while considering cognitive and genetic biomarkers.	For ADNI dataset: Accuracy: 99.2% Sensitivity: 98.9% Specificity: 99.5% For ADNI + Milan dataset: Accuracy: 98.2% Sensitivity: 98.1% Specificity: 98.3%
[40]	Alzheimer’s disease	Support vector machine	Supervised learning	Collected their own private dataset	Not pre-trained	This paper studied patients of Alzheimer’s in Thailand using SVMs and concluded that the hippocampus is a good classifier, producing high accuracy.	The subjects studied were not critically affected by Alzheimer’s. Additionally, the number of subjects used for this study was low and not sufficient enough for proper testing.	Accuracy: 62.64% On using clinical parameters: - Accuracy: 83–90%
[41]	Dementia	Support vector machine	Supervised learning	OASIS	Not pre-trained	By combining SVM with DA, good accuracy is obtained.	Large kernel scale reduces sensitivity.	Only SVM: Accuracy: 67.57% SVM with DA: Accuracy: 81.08% SVM with PCA: Accuracy: 70.27%
[42]	Dementia	Support vector machine	Supervised learning	OASIS	Not pre-trained	Low gamma values as well as high regularized values are exhibiting better results.	The classification of the subject groups is not precise in some cases.	Accuracy: Nearly 70% Sensitivity: 65–82%
[45]	Parkinson’s disease	K-means clustering	Unsupervised learning	Collected their own private dataset	Pre-trained	Clusters formed are given to the FOG detection system in a random order.	Sampling frequency was not high so it resulted in fewer data to be updated.	Accuracy: 93.2% Sensitivity: 92.4% Specificity: 94.9%
[46]	Alzheimer’s disease	K-means clustering	Unsupervised learning	OASIS	Not pre-trained	Top- and bottom-hat filtering is used to increase MRI image quality which was then fed to the system.	The Watershed method is not able to segment more objects.	N/A
[47]	Dementia	K-means clustering	Unsupervised learning	Collected their own private dataset	N/A	A combination of the sliding window approach, k-means clustering, and dynamic network analyses was used.	Patients on dopaminergic medication were not detected by the model.	N/A
[50]	Huntington’s disease	Decision tree	Supervised learning	NCBI GSE33000	Not pre-trained	A heterogeneous model, including random forest and rule induction models.	The mutant HTT gene may interfere with the promotion of Huntington’s disease pathogenesis.	Accuracy: 90.79 ± 4.57% Precision: 87.26 ± 6.95% Sensitivity/recall: 96.17 ± 3.30%
[51]	Alzheimer’s disease	Decision tree	Supervised learning	OASIS	Not pre-trained	Optimized using entropy and information gain.	Identification of symptoms to detect at an early stage.	Accuracy: 99.1%
[52]	Alzheimer’s disease	Decision tree	Supervised learning	OASIS	Not pre-trained	The model proposed was trained without fine tuning and then utilized the grid search to find the best possible parameters to fine tune.	Had a lower accuracy rate in comparison with other models.	Training accuracy: 100% Testing accuracy: 72% Test recall: 67%
[55]	Parkinson’s disease	Random forest	Supervised learning	NewHandPD	Not pre-trained	Combined with PCA, used to make a prediction based on handwritten data of the patients.	No general feature extraction method for different handwritings improved final voting performance.	Accuracy: 89.4% Specificity: 93.7% Sensitivity: 84.5% F1-Score: 87.7%
[56]	Parkinson’s disease	Random forest	Supervised learning	PPMI	Not pre-trained	Bootstrapping is performed where a random sample is taken from the available samples with replacement of data.	The decision trees might be open to overfitting.	Accuracy: 94.48%
[57]	Parkinson’s disease	Random forest	Supervised learning	Parkinson’s Dementia Clinical Epidemiology Data	Not pre-trained	Used explanatory variables that were randomly chosen from the samples.	A weighted voting system needs to be used to obtain a higher accuracy.	Accuracy: 65.6% Sensitivity: 70.6% Specificity: 60.0%
[60]	Dementia	Naïve Bayes	Supervised learning	Register based database of the Show Chwan health system	Not pre-trained	Multiplied the class prior probability with the likelihood of the disease in the datasets of the patients.	The study conducted may show selection bias.	Sensitivity: 92% Specificity: 95%
[61]	Alzheimer’s disease	Naïve Bayes	Supervised learning	OASIS	Not pre-trained	Processed MRI features are taken in partition vectors to feed the model.	Blood cell content, protein–protein and gene–gene interactions, etc., data were not incorporated.	Accuracy: 90%
[62]	Parkinson’s disease	Naïve Bayes	Supervised learning	-	Not pre-trained	Used a multi-feature evaluation approach.	May have some fluctuations that affect the final output.	Accuracy: 89.34% Recall: 89.3% Precision: 89.8%
[71]	Parkinson’s disease	Extreme learning machine	Hybrid of both supervised and unsupervised learning	Parkinson’s voice database	Pre-trained	Uses local binary pattern descriptors.	Not yet tested with databases of large-scale images.	Accuracy: 92.59%
[72]	Huntington’s disease	Extreme learning machine	Supervised learning	The HD dataset	Pre-trained	Imputes missing values using previously observed features.	The quality of prediction decreases as larger time intervals are taken into consideration.	F1 score: 91.9 ± 2.4%
[75]	Alzheimer’s disease	Co-training classification	Semi-supervised learning	ADNI	Not pre-trained	Uses multimodal neuroimaging data.	Various other co-training algorithms can be evaluated to improve the performance of the proposed system.	Accuracy: 92.91 ± 1.48% Sensitivity: 95.20 ± 1.63% Specificity: 90.70 ± 2.20%

N/A—Not Available.

**Table 2:** A summary of works on deep learning for degenerative nerve disease diagnosis.

Ref	Degenerative Nerve Diseases	Deep Learning Approach Used	Learning Model	Dataset Used	Pre-Trained or Not	Key Contribution	Limitations	Metrics
[79]	Alzheimer’s disease	Recurrent neural network	Supervised learning	NACC	Not pre-trained	An enhanced “many-to-one” RNN architecture is used to support the shift of time steps. This allows considering irregular visits of the patient.	The model is not equipped for early-stage predictions, which are important for beginning early treatment of the disorder.	Accuracy: 99.06 ± 0.43%
[85]	Alzheimer’s disease	Deep autoencoders	Unsupervised learning	ADNI	Not pre-trained	Generates accurate deviation maps and overcomes normative model variational autoencoders to estimate patient-level deviations with uncertainty estimates and also overcame the limitations of other approaches where subject-level deviations were found, which were supposed to be deterministic.	The generalizability of the approach is not yet validated.	N/A
[87]	Parkinson’s disease	Deep autoencoders	Unsupervised learning	PD dataset from database	Not pre-trained	Vocal impairments can be identified using sparse autoencoders.	Performance might not be the same if availability of data is not abundant.	Accuracy: 95% Sensitivity: 96% Specificity: 98%
[88]	Amyotrophic lateral sclerosis	Deep autoencoders	Unsupervised learning	PD dataset from UCI database	Not pre-trained	Uses deep autoencoders to denoise raw data.	Lack of availability of datasets decreases the scalability of the method.	Accuracy: 91.53% F1 score: 94.36%
[89]	Amyotrophic lateral sclerosis	Deep autoencoders	Unsupervised learning	PRO_ACT database	Not pre-trained	Uses stacked autoencoders to denoise raw available data.	Availability of better data must always be abundant to improve accuracy.	Accuracy: 87%
[94]	Parkinson’s disease	Deep belief network	Unsupervised learning	PD dataset from UCI database	Pre-trained	Hybrid of clustering and deep belief network was used with the aid of support vector regression. Self-organizing maps were also used to improve accuracy and scalability.	The dataset used had a limited number of features; hence, other datasets might show varied results. A supervised learning technique was used.	N/A
[97]	Alzheimer’s disease	Deep convolutional network	Supervised learning	OASIS	Pre-trained	Used a 12-layer CNN on brain MRI data to detect and classify Alzheimer’s disease.	Does not support multi-class classification.	Accuracy: 97.75% Demented Recall: 92% Non-demented Recall: 100% Demented Precision: 100% Non-demented Precision: 93% Demented F1 score: 97% Non-demented F1 score: 98%
[100]	Parkinson’s disease	Deep convolutional network	Supervised learning	Collected data on their own	Not pre-trained	Automating the process of diagnosis from continuous native speech with a relatively small set of training samples.	This model’s operation was considerably slow as it took longer training time.	Accuracy: 83.63%
[101]	Parkinson’s disease	Deep convolutional network	Supervised learning	HandPD	Not pre-trained	Extraction of features from various handwritings.	Slow speed of operations and large training time.	Accuracy: 78.18%
[102]	Amyotrophic lateral sclerosis	Deep convolutional network	Supervised learning	GWAS	Pre-trained	Use of CNN with vectors for predicting the recurring sequence of patterns in DNA and RNA binding proteins. The model also incorporated external domain knowledge, which aided in achieving higher performance.	The model assumed that the locus contains at least one casual variant; hence, the presence of false-positive GWAS signals may lead to significant loss in performance.	N/A

N/A—Not Available.

## Data Availability

Not Applicable.

## Appendix A

**Table A1:** List of Glossary/Nomenclature/Abbreviations used in the manuscript and their expansion.

Acronym	Full Form
MRI	Magnetic resonance imaging
EEG	Electroencephalography
SPECT	Single photon emission computerized tomography
IoT	Internet of Things
PRISMA	Preferred Reporting Items for Systematic Reviews and Meta-Analyses
DNA	Deoxyribonucleic acid
CAG	Cytosine, adenine, guanine
ALS	Amyotrophic lateral sclerosis
FXN	Frataxin
SMA	Spinal muscular atrophy
ANN	Artificial neural network
SVM	Support vector machine
DA	Dragonfly algorithm
FOG	Freezing of gait
LBD	Lewy body dementia
HPT	Hyper parameter tuning
OASIS	Outcome and assessment information set
CART	Classification and regression trees
PCA	Principal component analysis
KNN	K-nearest neighbor
LSTM	Long short-term memory
BRNN	Bidirectional recurrent neural networks
RNN	Recurrent neural networks
ADNI	Alzheimer’s Disease Neuroimaging Initiative
AD	Alzheimer’s disease
PET	Positron emission tomography
CT	Computed tomography
PD	Parkinson’s disease
DBN	Deep belief network
LDA	Linear discriminant analysis
ELM	Extreme learning machine
CNN	Convolutional neural network
DCNN	Deep convolutional neural network
HD	Huntington’s disease
ML	Machine learning
DL	Deep learning
AR	Augmented reality
VR	Virtual reality
AI	Artificial intelligence
DNN	Deep neural network
PNN	Probabilistic neural network
ROC	Receiver operating characteristic
